# Five-year Stability of Job Characteristics Scale Scores among a Japanese Working Population

**DOI:** 10.2188/jea.15.228

**Published:** 2005-11-07

**Authors:** Kazunori Kayaba, Akizumi Tsutsumi, Tadao Gotoh, Shizukiyo Ishikawa, Yoshihiko Miura

**Affiliations:** 1 Saitama Prefectural University, School of Health and Social Sciences.; 2 Okayama University Graduate School of Medicine and Dentistry.; 3 Jichi Medical School, Division of Community and Family Medicine, Center for Community Medicine.

**Keywords:** job strain, Karasek’s model, WHO-MONICA Psychosocial Study Questionnaire, long-term stability, population study

## Abstract

BACKGROUND: The job characteristics scale of job strain, which combines high job demands and low decision latitude based on Karasek’s model, has been applied to studies on health care and cardiovascular disease. However, little is known about the long-term stability of this scale with exposure of workers to job. We investigated the 5-year intraindividual variation in job characteristics scores among healthy community workers.

METHODS: Subjects of the study were 458 community dwelling persons forming part of the Jich Medical School Cohort Study at Yamato (currently, Minami-Uonuma city), Niigata prefecture. The Japanese version of the World Health Organization Multinational Monitoring of Trends and Determinants in Cardiovascular Disease (WHO-MONICA) Psychosocial Study Questionnaire was implemented twice (from 1992 through 1995, and in 1999) to measure job demands and decision latitude levels. Intraclass correlation coefficients were computed to evaluate stability of scores of the questionnaire.

RESULTS: Intraclass correlation coefficient of the decision latitude scores was 0.629 (95% confidence interval: 0.564 - 0.686) and that of the job demands scores was 0.551 (0.476 - 0.617). Subgroup analyses by age, sex, education level, years since first employment, number of co-workers, and job category and status at baseline revealed similar results. In contrast, subjects who experienced position changes within the same enterprise or changed jobs showed lower correlation coefficients of both decision latitude and job demands scores compared to those who experienced no change in job contents.

CONCLUSION: The Japanese version of the WHO-MONICA Psychosocial Study Questionnaire showed statistically significant long-term stability and could be to some extent responsive to change in job strain levels.

The job characteristics scale of job strain, which combines high job demands and low decision latitude based on Karasek’s model,^1^ has been applied to studies on health care and cardiovascular disease in North America, Europe and East Asian countries.^2^^,^^3^^,^^4^ Moreover, a number of studies^5^^,^^6^^,^^7^^,^^8^^,^^9^ have reported acceptable levels of reliability based on internal consistency using data at only one time point. The Japanese version^10^ of the World Health Organization Multinational Monitoring of Trends and Determinants in Cardiovascular Disease (WHO-MONICA) Psychosocial Study Questionnaire^11^ is one of the representative scales used for the Karasek’s model in Japan. An acceptable level of internal consistency has also been reported for this.^12^

In prospective studies, job strain levels at baseline have been regarded as representative of chronic levels and consequently used as long-term risk indicators. Although it is plausible that job characteristics change even within a job title and that employees develop coping skills against stressful situations deriving from such changes, few studies have reported the long-term stability of the job characteristics scale with exposure to job strain. We therefore investigated the 5-year intraindividual variation in job characteristics scores among healthy community workers in Japan.

## METHODS

This study formed part of the Jichi Medical School Cohort Study, a large-scale population-based prospective study designed to explore the risk factors for cerebro-cardiovascular disease in 12 Japanese communities. Local governments in all areas approved the study and informed consent was obtained from all participants. Details of the study design were published previously.^13^

**Figure 1.**
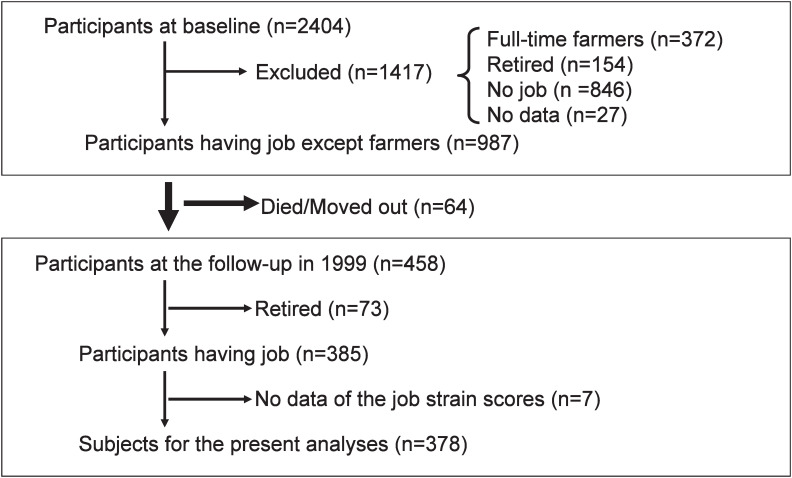
Outline of the subjects selection in the present analyses.

The cohort study population in the present analyses comprised residents from a community in Yamato (currently, part of Minami-Uonuma city), Niigata prefecture. Baseline data were collected from 1993 through 1995 with mass screening for cerebro-cardiovascular diseases conducted in accordance with the health and medical service law for the aged. Invitations to participate were sent to eligible individuals by the local government office of Yamato in accordance with the law; all residents aged 19 to 69 years were included. Those undergoing treatment or care for cardiovascular diseases was excluded from the cohort. A total of 2404 participants agreed to participate in the cohort study. For the present analyses, retired persons (154), full-time housewives (846), and subjects without job category data (27) were excluded from the participants. Full-time farmers (372) were also excluded from the analyses because little is known about validity and reliability of the job characteristics scale for Japanese farmers. Thus, 987 full- and part-time workers were eligible for potential subjects for the analyses; 95% worked for small-size enterprises employing ≤50 members of staff. All were followed-up annually, after collection of baseline data, by home visits, phone calls, mail, and interviews during annual health examinations. Of the 987 participants at baseline examination, 17 died and 47 dropped out because of moving. Four hundred and fifty eight (46.4 percent), 199 men and 259 women, attended the follow-up examination in 1999. The mean follow-up period was 5 years. The subject selection process for the present analyses is shown in Figure 1.

Sociodemographic and behavioral variables were investigated with a standardized questionnaire that was completed independently; answers were checked by a trained interviewer. The questionnaire consisted of the following items: occupation, status in the work place, years since first employment, number of co-workers, and changes in job content during the follow-up period. The following categories were included under the occupation item: security guard (n=3), service (104), transport (5), construction (77), production (132), merchant (60), clerk (28), and professional (49). The first five and last three categories were designated blue- and white-collar occupations, respectively.

The Japanese version of the WHO-MONICA Psychosocial Study Questionnaire^10^ was used to evaluate job strain levels at baseline examination (from 1992 through 1995) and follow-up in 1999. The questionnaire consists of two scales, decision latitude and psychological job demands. Decision latitude is defined as the sum of two subscales given equal weight: (a) skill discretion, measured by four elements (the continuous need to acquire new knowledge, skill requirements, creativity requirements, and repetitiveness [reversed score]), and (b) decision authority, measured by two elements (freedom to make decisions and choice in the approach to work). Higher scores indicate a higher level of decision latitude. Psychological job demands are defined by five elements (the need to work fast, the need to work hard, demands for extra work, insufficient time to do work, and conflicting demands). Higher scores indicate higher demand status. All questions were scored on a Likert scale of 1 to 4. The psychometric properties of the Japanese version of the demand-control questionnaire were reported previously.^14^^,^^15^ Cronbach’s coefficient alpha for the decision latitude and psychological demands scores were 0.80 and 0.79, respectively, with the baseline data obtained from the present subjects.

Descriptive parameters are shown as arithmetic means with standard deviations and percentage. The unpaired student’s t-test and chi-square test were used to compare the baseline characteristics of participants and those of non-participants at the follow-up examination. The stability of the job characteristics scale measurements at baseline and follow-up was evaluated by calculating intraclass correlation coefficients and their 95% confidence intervals (CIs) using the two-way mixed effects model with absolute agreement. Differences in scores between baseline and follow-up were tested using a paired t-test. A general linear model was used for comparisons between the no job change group and other groups. Significance was defined as p<0.05. All statistical analyses were performed using the SPSS^®^ statistical package 11.5j for Windows (SPSS, Chicago, Illinois, USA) with default settings.

## RESULTS

The baseline characteristics of individuals who participated in the 1999 follow-up and those who did not are shown in Table 1. Compared to the non-participants, participants were more likely to be women (56.6 vs. 49.3%, p<0.05) and to have a slightly higher job demands score (11.8±2.7 vs. 11.4±2.8, p<0.05). No statistically significant differences were observed with regard to age, education level, job-related variables, and decision latitude score.

**Table 1.** Baseline characteristics of the study population according to those who participated in the follow-up examination and those who did not.

	Follow-up examination:				p-value

	Participants		Non-participants		
No.	458*		529		
Sex, females (%)	259	(56.6)	261	(49.3)	<0.05
Age (years)**	46.7	11.4	45.2	12.9	n.s.
Education status (%)					n.s.
Elementary and junior high school	248	(54.4)	288	(55.2)	
High school	154	(33.8)	167	(32.0)	
University or other	54	(11.8)	67	(12.8)	
Years since first employment (%)					n.s.
Quartile 1 (12-15 years)	95	(20.9)	103	(19.6)	
Quartile 2 (16-17 years)	117	(25.7)	121	(23.0)	
Quartile 3 (18-19 years)	141	(31.0)	168	(31.9)	
Quartile 4 (≥20 years)	102	(22.4)	134	(25.5)	
No. of co-workers (%)					
Quartile1(1-2)	136	(30.6)	123	(24.8)	n.s.
Quartile2(3-5)	122	(27.4)	141	(28.4)	
Quartile3(6-10)	103	(23.1)	120	(24.2)	
Quartile4(≥11)	84	(18.9)	112	(22.6)	
Job category (%)					n.s.
White-collar occupations	137	(29.9)	176	(33.3)	
Blue-collar occupations	321	(70.1)	353	(66.7)	
Status at work (%)					n.s.
Administrative	70	(15.5)	91	(17.5)	
Non-administrative	383	(84.5)	430	(82.5)	
Job strain scores**					
Decision latitude^†^	15.6	±3.2	15.7	±3.4	n.s.
Psychological job demands^‡^	11.8	±2.7	11.4	±2.8	<0.05

* : This includes a number of subjects retired.

**: Values represent the mean ± standard deviation.

† : Higher scores indicate higher levels of decision latitude.

‡ : Higher scores indicate higher levels of demand.

n.s. not significant

Of the 458 participants of the follow-up in 1999, 73 retired during the follow-up period. A further 7 were excluded because of missing job strain score values at follow-up examination. Data of a total of 378 workers were therefore analyzed.

**Figure 2.**
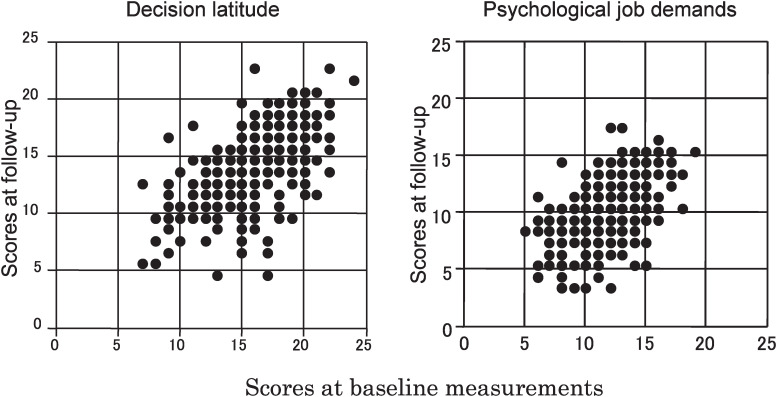
Correlation of decision latitude and job demand scores between baseline and follow-up. Whole subjects.

Intraclass correlation coefficient of the decision latitude scores was 0.629 (95% CI: 0.564 to 0.686) and that of the job demands scores was 0.551 (95% CI: 0.476 to 0.617) (Table 2 and Figure 2). Subgroup analyses according to age, sex, education level, years since first employment, number of co-workers, and job category and status at baseline revealed similar results (Table 2). A high correlation coefficient for decision latitude score was observed for workers with a higher education level (0.894, 95% CI: 0.818 to 0.939). Correlation coefficients for scores at baseline and follow-up examinations of decision latitude scale and of job demands scale among the 63 subjects who experienced position changes within the same enterprise or changed jobs were lower than those for subjects who experienced no changes (Table 2).

**Table 2.** Intraclass correlation coefficients (ICC) of decision latitude and job demands scores between baseline and 5-year follow-up.

	Decision latitude			Job demands		
	
	N*	ICC	95% confidence interval	N	ICC	95% confidence interval
Whole subjects	377	0.629	0.564 - 0.686	378	0.551	0.476 - 0.617
Age(year)						
19-29	32	0.503	0.186 - 0.723	32	0.415	0.076 - 0.666
30-39	92	0.678	0.546 - 0.777	92	0.608	0.461 - 0.722
40-49	130	0.674	0.568 - 0.758	129	0.531	0.394 - 0.644
50-59	76	0.556	0.378 - 0.694	76	0.595	0.429 - 0.722
60-69	47	0.567	0.335 - 0.734	49	0.532	0.298 - 0.706
Sex						
Men	181	0.582	0.476 - 0.670	182	0.612	0.513 - 0.696
Women	196	0.587	0.487 - 0.671	196	0.410	0.286 - 0.520
Education status						
Elementary or Junior High school	191	0.593	0.492 - 0.677	192	0.572	0.469 - 0.660
High school	137	0.561	0.434 - 0.665	137	0.457	0.314 - 0.579
University or other school	48	0.894	0.818 - 0.939	48	0.661	0.466 - 0.794
Years since first employment (year)						
Quartile1 (12-15)	79	0.600	0.438 - 0.724	79	0.525	0.344 - 0.669
Quartile2 (16-17)	100	0.619	0.482 - 0.727	99	0.625	0.488 - 0.731
Quartile2 (18-19)	123	0.580	0.450 - 0.686	123	0.425	0.268 - 0.560
Quartile4 (20-)	74	0.736	0.611 - 0.825	76	0.629	0.470 - 0.748
Number of co-worker						
Quartile1(1-2)	120	0.478	0.327 - 0.605	119	0.575	0.441 - 0.684
Quartile2(3-5)	103	0.586	0.443 - 0.699	103	0.571	0.425 - 0.688
Quartile3(6-10)	85	0.704	0.579 - 0.797	86	0.651	0.510 - 0.757
Quartile4(≥11)	59	0.646	0.468 - 0.774	59	0.349	0.104 - 0.554
Job category						
White-collar occupations	118	0.632	0.510 - 0.729	117	0.549	0.409 - 0.664
Blue-collar occupations	259	0.622	0.542 - 0.691	261	0.543	0.451 - 0.623
Job status						
Administrative	63	0.553	0.356 - 0.703	64	0.574	0.384 - 0.717
Non-administrative	310	0.595	0.517 - 0.663	310	0.513	0.426 - 0.590
Change of job contents						
No change	315	0.678	0.613 - 0.733	316	0.620	0.548 - 0.684
Position change	22	0.195	-0.182 - 0.545	23	0.170	-0.210 - 0.523
Job change	40	0.431	0.151 - 0.649	39	0.302	-0.021 - 0.561

* : For some variables, the numbers do not total 378 because of missing values.

Decision latitude scores tended to increase after position (mean difference: 1.6, 95% CI: -0.1 to 3.3) and job changes (0.9, 95%CI: -0.1 to 1.9), although these results were not statistically significant. No meaningful interpretations were found with regard to changes in job demands scores.

To explore bias due to the healthy worker effect, decision latitude and job demands scores at baseline were compared according to job change status, including retirement, using a multivariate generalized linear model. After adjusting for age, the no job change group showed a statistically significant higher decision latitude score at baseline than other groups (Table 3).

**Table 3.** Age-adjusted scores of the decision latitude and the job demands scale at the baseline examination by the job status changes.

Job change status	N	Adjusted mean*			SE	Estimated difference*			95% confidence interval				

							Decision latitude score						p<0.0001

No change	316	16.2			3.1			Reference					
Position change	23	14.4			2.5			-2.0	-3.3		-	-0.7	
Job change	40	14.5			3.1			-1.9	-2.9		-	-0.9	
Retired	72	13.8			3.0			-2.2	-3.0		-	-1.3	


							Job demands score						p=0.064

No change	317	12.0			2.5			Reference					
Position change	24	10.8			3.0			-1.1	-2.2		-	0.0	
Job change	39	11.4			2.9			-0.6	-1.4		-	0.3	
Retired	71	11.5			3.0			-0.7	-1.4		-	0.1	

* : Estimated with multivariate generalized linear model.

SE: standard error of the mean

## DISCUSSION

Of the workers who participated in both baseline and follow-up examination, 84% did not change their job or job status during the study period, and their job strain scores showed moderate correlation coefficient levels. Results varied with age, sex, education status, years since first employment, and number of co-workers, and job category and status at baseline. Subgroups that experienced changes showed lower coefficients and the decision latitude scores at follow-up examination tended to be higher than those at baseline.

To the best of our knowledge, this is the first study to evaluate the long-term reproducibility of the job characteristics scale in a large-scale population-based study in Japan. Two US studies previously reported the reproducibility of this scale. As part of the Work Site Blood Pressure Study in New York City, the 3-year test-retest correlation coefficient was shown to be 0.64 for job decision latitude and psychological job demands.^6^ A four-year follow-up study of female nurses showed a moderate degree of stability with correlation coefficients for job control and job demands of 0.60 and 0.54, respectively.^16^ These results are similar to those obtained here.

When compared with other behavioral factors, the coefficients for leisure-time physical activity in a Finnish study ranged from 0.41 to 0.43.^17^ Regarding biomedical factors in the present population, we previously reported^18^ that the coefficients for job strain scores were lower than those for body mass index (0.93), total and high-density lipoprotein cholesterols (0.73 and 0.75, respectively), and blood pressure (0.65). The job characteristics scores in our study seemed to have similar reproducibility compared with other behavioral and biomedical factors in the population-based studies.

Education is known as an important determinant of workers’ health.^19^ Higher correlation coefficients were observed for those with higher educational attainment. This is probably because they would have achieved a more stable job position than those with lower education levels.

Workers who experienced changes in their job or job status showed a weaker correlation between baseline and follow-up job strain scores and their decision latitude levels tended to increase. Although the small number of subjects who experienced job changes limits interpretation of the results, the job characteristics scales adopted seem responsive to change.

While some studies in Europe and North America have shown a significant relationship between high job strain and ischemic heart disease,^1^ considerable numbers of studies, including a study of Japanese immigrants in Hawaii, have failed to show a significant positive association.^20^ One reason is suggested to be misclassification of job characteristics due to the lack of information on cumulative exposure to high job strain.^2^^,^^21^ Instability of job strain levels during study periods could also result in underestimations of the association between job characteristics and health problems. Our results estimating the responsiveness of the job characteristics scale to changes in job strain levels could support this partly.

In previous studies examining the relationship between behavioral work characteristics and health, subjects have mainly included workers from large enterprises with a narrow job category range. Studies on employees in small-sized firms, who tend to have diverse job categories, are scarce.^2^ Moreover, the annual statistics of the Japanese labor force survey^22^ reported that 97% of enterprises employ ≤50 members of staff, and 62% of the total work force work for small enterprises with ≤50 employees. In this study, 95% of the subjects worked for small-size enterprises (≤50 employees), and 69% were categorized as blue-collar workers. Our findings are unique in that they were derived from workers with diverse occupations, and therefore they are valuable with regard to Japanese workers’ health.

This study has some limitations that need to be addressed in future research. First, most subjects in the present analysis were middle aged with relatively high levels of job security. The results displayed in Table 3 suggest that most subjects developed job adaptation skills prior to the baseline examination. This healthy workers effect could have biased our results. Secondly, the small number of subjects who experienced changes in their job circumstances lowered the power to detect the responsiveness of score changes. Thirdly, categorization of workers into job groups defined on the basis of self-administered questionnaire scores could induce misclassification. This study probably underestimated differences of stability of the job strain scores between job categories. Finally, the results of this study were restricted to information from workers living in a local municipality and therefore we should be cautious in generalizing the study findings to other municipal urban populations.

Despite these limitations, however, the findings of this study have important implications. The Japanese version of the WHO-MONICA Psychosocial Study Questionnaire showed statistically significant long-term stability and was supposed to be to some extent responsive to change in job strain levels.
